# A Giant Gallstone: The Largest Gallstone Removed Laparoscopically in the World

**DOI:** 10.7759/cureus.7933

**Published:** 2020-05-02

**Authors:** Yardesh Singh, Sidiyq Mohammed, Aqilah Hosein, Kercheval Ramoutar, Vijay Naraynsingh

**Affiliations:** 1 Surgery, University of the West Indies, St. Augustine, TTO; 2 Surgery, San Fernando General Hospital, San Fernando, TTO; 3 Surgery, Medical Associates Hospital, St. Joseph, TTO; 4 Clinical Surgical Sciences, University of the West Indies, St. Augustine, TTO

**Keywords:** gallstone, mis, laparoscopic cholecystectomy, giant gallbladder

## Abstract

Trinidad and Tobago, a small twin island republic off the coast of Venezuela, is leading the Caribbean in laparoscopic surgery. While giant gallbladders are usually difficult to operate on and have a high conversion rate from laparoscopic to open procedure, in Trinidad and Tobago a laparoscopic cholecystectomy involving a giant gallbladder and the largest gallstone ever removed laparoscopically was performed uneventfully.

## Introduction

Gallstone disease is very prevalent in the Western hemisphere and is becoming increasingly prevalent in the East [1]. The Caribbean, and in particular Trinidad and Tobago, is no exception, as is evident at the San Fernando General Hospital, where many laparoscopic cholecystectomies are performed on a yearly basis [2].

We present the case of a cholecystectomy and, to the best of our knowledge following a thorough literature review, the removal of the largest gallstone done laparoscopically in the world.

## Case presentation

**Figure 1:**
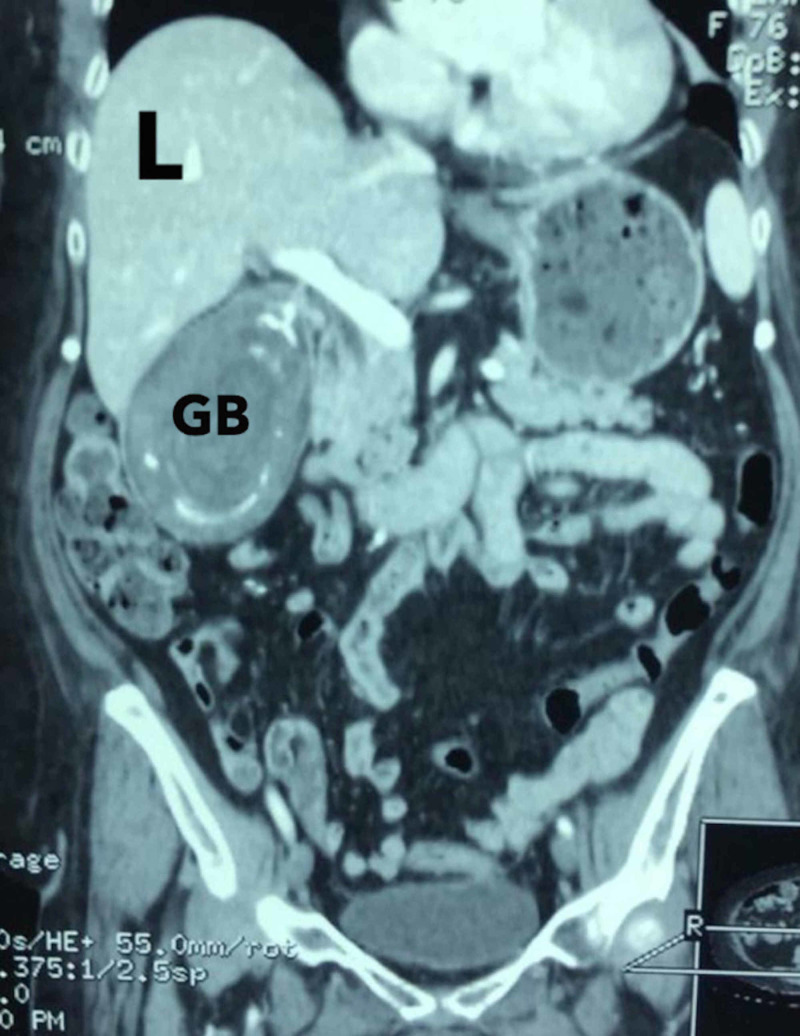
CT scan showing a giant gallbladder (GB) occupied by a solitary gallstone just inferior to the liver (L)

A 72-year-old female presented with features of biliary colic ongoing for one year, noted after ingestion of a fatty meal. On examination, she was found to have a mildly painful, palpable gallbladder, but no jaundice. Her blood panel was unremarkable. On contrast CT scan of the abdomen, a 14 cm x 7 cm solitary mass was noted within the gallbladder, postulated to be a gallstone (Figure 1).

**Figure 2:**
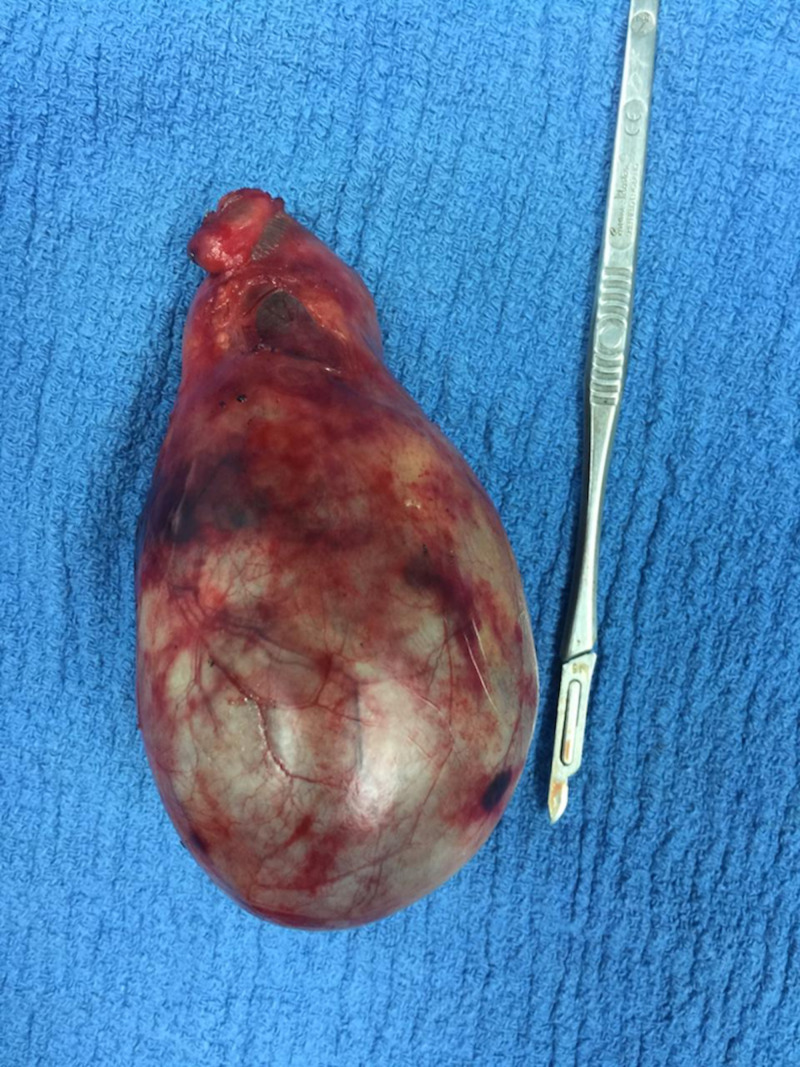
Intact giant gallbladder post laparoscopic cholecystectomy

**Figure 3:**
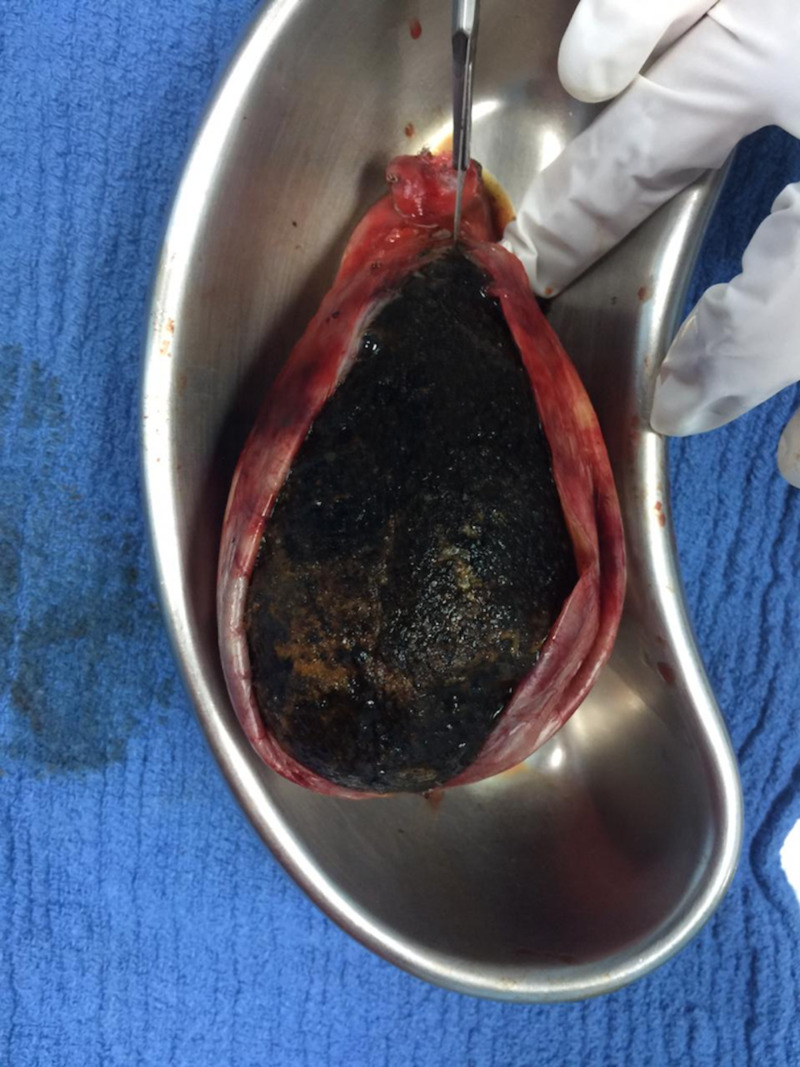
Giant gallbladder opened to reveal a solitary, pear-shaped gallstone

**Figure 4:**
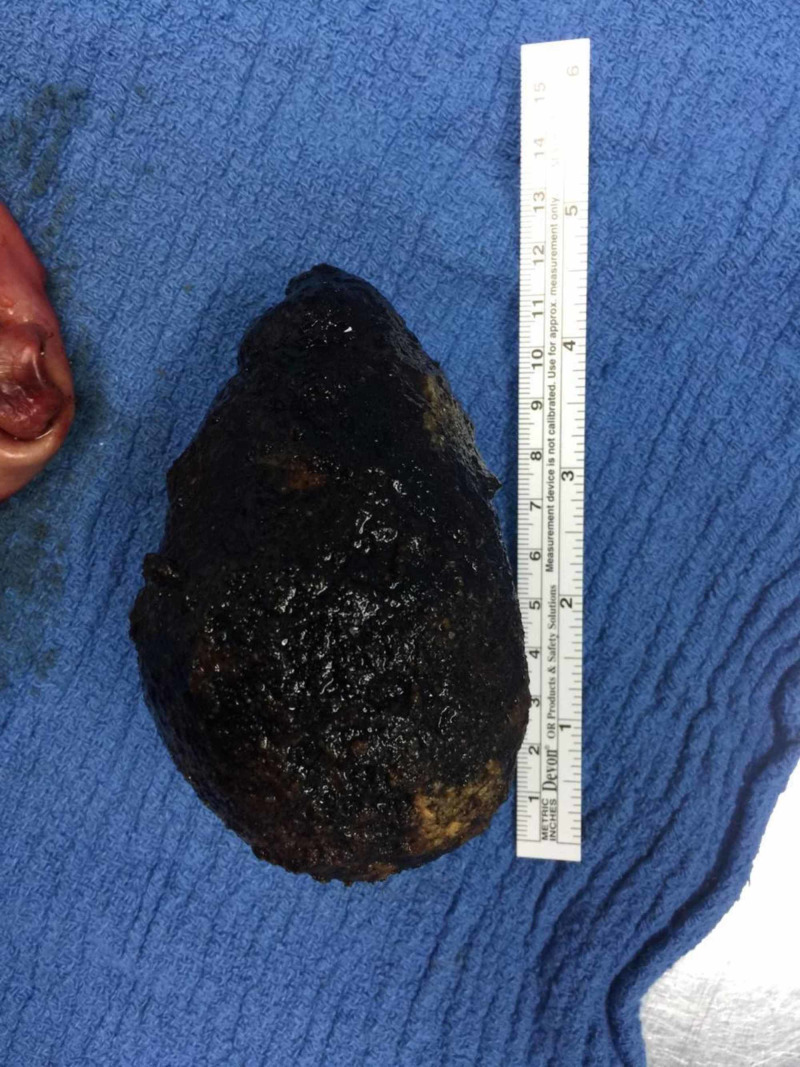
Giant gallstone measuring 12.8 cm in length

At surgery, the huge gallbladder was fully distended, and much surgical dexterity had to be employed to manipulate this gallbladder during the operation. The cholecystectomy was successfully performed using routine port placements; the umbilical incision site through which the camera port was placed was lengthened to just over 7 cm in order to safely deliver the gallbladder and its giant gallstone intact (Figures 2-4). The postoperative course was uneventful; the patient was discharged the next day and has been asymptomatic for one year. She gave full consent for her case presentation and pathology to be used for publication and education purposes.

## Discussion

Laparoscopic cholecystectomy is the gold standard treatment for gallstone disease and can be achieved in 96% of the cases; the rate of conversion from laparoscopic to open cholecystectomy is about 4%-5%. The gallstone with a diameter of over 5 cm is very rare, and such cases prove to be difficult to attempt laparoscopically. Some surgeons may even consider a giant gallstone an indication for a classical cholecystectomy [3]. The risk of conversion is related to surgeon factors, patient factors, and possibly equipment factors. In this instance, the cholecystectomy was performed uneventfully.

During a cholecystectomy, giant gallstones may lead to conversion from laparoscopic to open procedure in two different ways. First, larger stones in the gallbladder can result in a thicker gallbladder wall secondary to inflammation. The thickness of the gallbladder wall has been shown to correlate with conversion to open surgery in bivariate analyses [4]. Second, a large gallstone can lend to technical difficulties during the surgery, for example, grasping the gallbladder with laparoscopic instruments may prove to be extremely difficult. In addition, the larger the gallbladder the more challenging it will be to gain appropriate anatomical exposure for conventional and safe dissection.

This operation was performed using standard port placements, and the total time of the procedure was approximately 67 minutes. The lead surgeon carried out all the steps in keeping with a routine laparoscopic cholecystectomy, with gentle but good retraction to expose the critical view [5]. There was difficulty in grasping the fundus of the gallbladder, and superior retraction was achieved by pushing the gallbladder using the opened jaws of a large grasper. The infundibulum was then grasped to provide traction to dissect and expose Calot's triangle. The peritoneal attachment was opened along the lateral aspect of the gallbladder, followed by medial dissection to identify the cystic duct and artery, before clipping and dividing the artery and duct. Removal of the gallbladder from the liver bed was done in a routine fashion. The middle port or camera port incision was lengthened to just above 7 cm in order to remove the specimen, and the fascia was closed primarily with a heavy, non-absorbable, monofilament suture. The traditional subcostal Kocher incision is infamous for its associated complications such as increased postoperative pain, splinting on inspiration, and resultant atelectasis. As such, the decision was made to extend the camera port incision for specimen retrieval in order to circumnavigate the potential complications of the subcostal incision. This proved beneficial as the patient had an uneventful postoperative course and was discharged one day after the operation. A 12.8 cm x 7 cm gallstone was found within this huge gallbladder, which seems to be the largest gallstone to be removed laparoscopically in the world. While Xu et al. detailed the laparoscopic retrieval of a 9.5-cm gallstone, and Becerra et al. reported removal of a 16.8-cm long gallstone via classical cholecystectomy in the emergency setting, from the literature to date, our gallstone appears to be the largest removed laparoscopically [6,7].

Gallstones are categorized into three types: pure cholesterol stones, pigment stones, and mixed stones. This stone was determined to be a cholesterol stone, the most common type of gallstone retrieved [8].

The formation of gallstones results from an irregularity in the normal relationships between the major constituents of bile: cholesterol, phospholipids, and bile acids. There are three main steps in gallstone formation: saturation, crystallization, and growth. In cases where there is a high cholesterol saturation index, cholesterol saturated vesicles are formed and initiate the nucleation of cholesterol monohydrate crystals which subsequently give rise to the core of a cholesterol stone. Chronic cholecystitis is an important factor in the overall chain of events, as it leads to mucin hypersecretion and rapid formation of cholesterol crystals [9]. There is no evidence that suggests that the pathogenesis of giant gallstones is different from the formation of regular-sized cholesterol stones.

## Conclusions

Although the conversion rate for giant gallbladders seems to be high, with good surgical experience larger and more complex laparoscopic cholecystectomies are becoming safer and easier to perform. In the hands of a proficient laparoscopic surgeon, even challenging cases such as this, with the largest gallstone removed laparoscopically in the world, can be executed with good outcomes.
